# Diffraction Line Broadening Analysis if Broadening Is Caused by Both Dislocations and Limited Crystallite Size

**DOI:** 10.6028/jres.109.005

**Published:** 2004-02-01

**Authors:** J.-D. Kamminga, L. J. Seijbel

**Affiliations:** Netherlands Institute for Metals Research, Rotterdamseweg 137, NL-2628 AL Delft, The Netherlands; Bruker Nonius BV, Oostsingel 209, NL-2612 HL Delft, The Netherlands

**Keywords:** crystallite size, diffraction line broadening, dislocations, nickel, recrystallization, x-ray diffraction

## Abstract

The determination of dislocation distribution parameters is discussed for specimens where both strain broadening caused by dislocations and size broadening occur. If the strain broadening is well described by a model due to Wilkens, several methods are possible for the analysis of the broadening of diffraction lines. In sputter deposited nickel layers, three different methods for diffraction line broadening analysis yield identical results. The recrystallization of the nickel layers was investigated by annealing the layers at various temperatures in the range 300 K to 500 K. With increasing annealing temperature, the microstructure of the layers changed from a microstructure with small grains and high dislocation density, via a microstructure that is a mixture of small grains with high dislocation density and large grains with low dislocation density, to a microstructure with large grains and low dislocation density.

## 1. Introduction

Since long, x-ray diffraction line broadening is used for the investigation of dislocation distributions; due to the stress fields induced by the dislocations atoms are displaced from their ideal lattice positions, which causes diffraction line broadening. Although theoretical models [1–3] as well as experimental equipment necessary to measure dislocation distribution parameters from diffraction line shapes have been available for several decades, the determination of dislocation distribution parameters in practice can still be problematic.

In part this is due to the simple dislocation distributions that underlie the theoretical models describing the diffraction line broadening in terms of dislocation distribution parameters. For example, in most cases dislocations are supposed to be straight and infinitely long, and to be distributed in a rather ideal way. The assumed simple dislocation distributions limit the reliability of the dislocation distribution parameters (as the dislocation density) determined in practice. Still, very detailed information can be obtained without precise quantitative knowledge of these parameters. For example, the type and orientation of the dislocations can be determined in practice (see, e.g., Refs. [4–6]). Such information is not easily assessed with other experimental techniques.

A second difficulty for the analysis of diffraction line shapes in terms of dislocation distribution parameters is of experimental nature. In general, next to the (strain) broadening due to dislocations, diffraction line broadening also occurs because of other lattice defects such as stacking and twin faults, a non-ideal instrument (instrumental broadening), and the limited size of the crystallites in the specimen (size broadening). For an interpretation of the diffraction line broadening in terms of dislocation distribution parameters, these additional broadenings should be taken into account in the analysis.

The present paper concerns this last problem. The paper focuses on the separation of size and strain broadening. Different methods of analysis are proposed and compared with each other and with methods of size strain separation known from the literature. As a prerequisite it is supposed that the strain broadening is well described according to the simple model due to Wilkens [1]. It is shown that in that case, the Warren-Averbach method [7] for the separation of size and strain broadening should not be applied. On the other hand, a method that is not often used [8] is particularly useful.

The methods discussed use two orders of a reflection measured at identical specimen orientations. In this respect the methods differ from the recently developed whole powder pattern fitting (WPPF) procedures [9,10], in which more reflections in the powder pattern are used for the analysis. In these methods either the Fourier coefficients, or diffraction line profiles themselves are calculated using models for the strain and size distributions in the specimens. This differs from the approach in this paper, because in the presently discussed methods a model for the size broadening is not used. A concern for the WPPF methods is that different diffraction peaks stem from different crystallites. Therefore, in the analysis, assumptions concerning the uniformity of the crystallites, the size of the crystallites, or the dislocation distributions therein, are made (see Ref. [11]). Such assumptions are not made in the methods in this paper because the two orders of reflections stem from the same crystallites. Therefore, the methods are also insensitive to texture. However, as compared to the information obtained from WPPF procedures, the information obtained is limited since only a small fraction of the crystallites is considered.

Strain broadening that cannot be written according to the model of Wilkens is not treated in the paper. Consequently, if, e.g., stacking and/or twin faults cause significant diffraction line broadening (the expressions due to Wilkens do not hold for broadening induced by faulting [12]), the methods of analysis discussed in the paper are not useful.

As an example of the analysis methods discussed, the recrystallization in thin nickel layers was investigated. The development of the dislocation distribution and grain size was assessed using x-ray diffraction measurements on specimens that were annealed at different temperatures. Such, a detailed picture of the recrystallization in the thin layers was obtained.

## 2. Background

### 2.1 Representation of Diffraction Line Profile in Fourier Coefficients

The intensity distribution *P*(*S*) of a structurally broadened diffraction line profile can be expressed as a Fourier series (e.g., Ref. [7]):

$$P(S)=K\sum_{L=-\infty }^{\infty }A(L)\cos(2\pi \;LS)+B(L)\sin(2\pi \;LS),$$ (1)

where *K* is approximately constant for a given diffraction profile and *A*(*L*) and *B*(*L*) denote the cosine and sine Fourier coefficients, respectively, belonging to correlation distance *L*. Theoretically *L* takes only discrete values, but in practice *L* can be considered as a continuous variable. *S* is the diffraction vector, which is related to the diffraction angle 2*θ* and the wavelength *λ* by *S* = 2sin*θ*/*λ*. Structural diffraction line broadening is often subdivided into size broadening, and strain broadening. The size coefficient *A*^S^(*L*) (the superscript S denotes size broadening) equals *N_L_*/*N*, where *N* denotes the number of unit cells in the specimen considered, and *N_L_* is the number of unit cell pairs at mutual distance *L*, where the distance *L* is taken parallel to the diffraction vector [1,8]. Size broadening is always symmetric (i.e., *B*^S^(*L*) = 0). The total structurally broadened profile is the convolution of the size-broadened profile and the strain-broadened profile. In terms of Fourier coefficients (the superscript D denotes distortion (strain) broadening):

$$A(L)=A^{S}(L)A^{D}(L),$$ (2)

$$B(L)=A^{S}(L)B^{D}(L).$$ (3)

### 2.2 Strain Broadening Due to Dislocations: Wilkens Model

The Wilkens model for diffraction line strain broadening due to dislocations presupposes that in the diffracting crystallites so-called restrictedly random dislocation distributions are present [1]. In a restrictedly random distribution, all dislocations are infinitely long and straight. All dislocations belong to the same set: i.e., all dislocations are of the same character and belong to the same slip system (the dislocations are parallel). A cross-section of the crystal, normal to the dislocation lines, can be subdivided into sub-areas of equal size and shape containing exactly the same number of dislocations. Within each sub-area the dislocations are randomly distributed. Equal numbers of dislocations with positive and negative Burgers vectors are present in each sub-area: the net Burgers vector equals zero. Thus, the distribution is fully determined by the number of dislocations in each sub-area, as characterised by the dislocation density *ρ*, and the size of the sub-areas, which will be represented by the so-called outer cut-off radius *R*_e_ (see Refs. [1,5,13]). For circular sub-areas it can be shown: *R*_e_ ≈ 0.78*R* [1], where *R* is the radius of the circular sub-area.

The following has been derived for the strain cosine Fourier coefficient of the diffraction line profile broadened due to a restrictedly random distribution of dislocations [1,5]:

$$A^{D}(L)=e^{-PL^{2}(Q-\ln\;L)}$$ (4)

$$P=\frac{\pi }{2}g^{2}b^{2}C\rho$$ (5)

$$Q=\ln\;R_{e}+2\;\ln\;2-\frac{1}{3}-\ln\;\sigma \left|\mu \right|$$ (6)

where *L* is the correlation length, *b* is the length of the Burgers vector ***b***, *σ* equals |sin *ψ*| where *ψ* is the angle between the line vector *l* of the dislocation and the vector ***g***, where ***g*** is the diffraction vector at which Bragg's law holds exactly for the reflection considered (length *g*; for cubic material it holds: *g* = (*h*^2^ + *k*^2^ + *l*^2^)^1/2^/*a*_0_, where *a*_0_ is the lattice parameter of the specimen). The scalar product of ***g*** and ***b*** is denoted by *μ*. *C* is the so-called contrast factor for the particular dislocation type, slip system and reflection (see, e.g., Ref. [12]). The above expressions yield a proper description of the line profile if $R_{e}\sqrt{\rho }\geq 1$ and *L* ≤ 0.5*R*_e_/*σ* |*μ*| [1]. Further, the Wilkens model assumes symmetric line profiles (correctly; see Ref. [14]) and therefore the sine coefficients of the line profile are zero.

In practice, more than one set of dislocations will be present in a crystal. It was proposed in Ref. [1] that the diffraction line profile from a crystal with various sets of dislocations is the convolution of the line profiles from the single sets. Thereby, the elastic interaction of the various sets is neglected. Then, if more than one set *i* of dislocations is present Eq. (4) can still be used with (see Ref. [5]):

$$\bar{P}=\sum_{i}P_{i}=\frac{\pi }{2}g^{2}b^{2}\sum_{i}C_{i}\rho_{i},$$ (7)

$$\bar{Q}=\frac{\sum_{i}P_{i}Q_{i}}{\sum_{i}P_{i}}=2\;\ln\;2-\frac{1}{3}-\frac{\sum_{i}C_{i}\rho_{i}\ln\frac{\sigma_{i}\left|\mu \right|}{R_{e,i}}}{\sum_{i}C_{i}\rho_{i}},$$ (8)

It was shown in Ref. [14], that the elastic interaction between various sets is small.

## 3. Application to Experimental Data

In practice, the determination of defect distribution parameters from experimental data using the above expressions can be troublesome. The parameters $\bar{P}$ and $\bar{Q}$ must be extracted from a diffraction line profile containing, apart from strain broadening, instrumental broadening and size broadening. If the values of $\bar{P}$ and $\bar{Q}$ have been determined, they must be interpreted in terms of defect distribution parameters.

### 3.1 Removal of Instrumental Broadening

Instrumental broadening can be removed from an experimental profile if a reference specimen, containing negligible size and strain broadening, is available. For each reflection measured, a peak profile of the reference specimen, using identical diffractometer settings, must be recorded. After subtraction of the background for both peaks, their Fourier coefficients are determined. The Fourier coefficients of the peak without instrumental broadening then follow by dividing the Fourier coefficients of the broadened peak by the Fourier coefficients of the peak of the standard specimen (see, e.g., Ref. [7]). In the present case, because the structural broadening is symmetric, the moduli of the Fourier coefficients can be used. After removal of the instrumental broadening, Fourier coefficients are scaled such that the first coefficient, i.e., the coefficient for *L* = 0, equals 1.

For the deconvolution procedure discussed above the quality of the reference specimen is of paramount importance. Furthermore, peak overlap of other diffraction lines with the line profiles under investigation hinders the description of peak shape in terms of Fourier coefficients, because then the peak shape of the diffraction line under consideration must be extracted first. Relatively long tails must be measured for accurate background determination, because a small error in the background determination leads to an error in the first few Fourier coefficients (low *L*). At high *L* errors in the Fourier coefficients occur due to limited counting statistics and the finite step size used in the data acquisition. Therefore, relatively high counting time and a relatively small step size are required.

### 3.2 Dealing With Size Broadening

Various methods exist that separate the contributions of the size and the strain from diffraction line profiles. Several methods require measurements of two orders of a reflection at a given specimen orientation and assume that in both measurements the same crystallites are diffracting (defocusing of the diffractometer is thereby neglected). Consequently the size broadening is identical for both diffraction peaks. However, the strain broadenings are different for the different orders of reflection. Obviously, information about the non-diffracting crystallites is not obtained. Below, methods are discussed that can be used to separate size and strain broadening using two orders of reflection. Two important relations for the first and the second order reflections follow from Eqs. (7) and (8);

$${\bar{P}}_{2\text{nd}}/{\bar{P}}_{1\text{st}}=4$$ (9)

$${\bar{Q}}_{1\text{st}}-{\bar{Q}}_{2\text{nd}}=\ln\;2$$ (10)

(the order is indicated by a subscript). These expressions are used throughout the derivations of the expressions below.

If the Fourier coefficients of two line profiles containing exactly the same size broadening are divided, the size coefficients cancel. If Eqs. (4), (9), and (10) hold for the strain broadening, it follows for the ratio of the Fourier coefficients of the second order and first order diffraction line profiles [using Eq. (2)]:^1^

$$\frac{A_{2\text{nd}}(L)}{A_{1\text{st}}(L)}=\frac{A_{2\text{nd}}^{D}(L)}{A_{1\text{st}}^{D}(L)}=\exp\left[-3{\bar{P}}_{1\text{st}}L^{2}({\bar{Q}}_{1\text{st}}-\frac{4}{3}\ln\;2-\ln\;L)\right].$$ (11)

Consequently, ${\bar{P}}_{1\text{st}}$ and ${\bar{Q}}_{1\text{st}}$ (and thereby ${\bar{P}}_{2\text{nd}}$ and ${\bar{Q}}_{2\text{nd}}$) can be readily obtained by fitting Eq. (11) to the ratio of the Fourier coefficients obtained experimentally. Thereby, the strain coefficients are known. Then, the size coefficients are obtained, dividing the measured Fourier coefficients by the strain coefficients obtained.

In practice, several problems may arise using this method. First, the ratio is liable to experimental errors if *A*_1st_ is close to zero. Second, the expressions for the line profiles are only reliable for relatively small correlation distance. If *R*_e_ is small, this may lead to problems connected to a small fitting range, especially for the second order Fourier coefficients (and consequently for the ratio of the first and second order Fourier coefficients).

A better fitting range can be obtained using a method for the separation of size and strain broadening proposed in Ref. [8]. For strain Fourier coefficients for which Eqs. (4), (9), and (10) hold, $A_{1\text{st}}^{D}(L)=A_{2\text{nd}}^{D}\left(\frac{1}{2}L\right)$. From this result it follows:

$$\ln\left[A_{1\text{st}}\left(L\right)\right]-\ln\left[A_{2\text{nd}}\left(\frac{1}{2}L\right)\right]=\ln\left[A^{S}\left(L\right)\right]-\ln\left[A^{S}\left(\frac{1}{2}L\right)\right].$$ (12)

The term on the right-hand side of Eq. (12) can be approximated using $\ln\left[A^{S}\left(\frac{1}{2}L\right)\right]\approx \frac{1}{2}\ln\left[A^{S}\left(L\right)\right]$, which holds for small *L* [8]. Consequently, the size Fourier coefficients can be obtained from:

$$\ln\left[A_{1\text{st}}\left(L\right)\right]-\ln\left[A_{2\text{nd}}\left(\frac{1}{2}L\right)\right]=\frac{1}{2}\ln\left[A^{S}\left(L\right)\right].$$ (13)

In the following, the determination of the size coefficients using Eq. (13) will be referred to as the Van Berkum-Vermeulen (VB-V) analysis. The strain coefficients of the first and second order reflection are determined, dividing the measured Fourier coefficients by the size coefficients [cf. Eq. (2)]. Values of ${\bar{P}}_{1\text{st}}$, ${\bar{P}}_{2\text{nd}}$, ${\bar{Q}}_{1\text{st}}$, and ${\bar{Q}}_{2\text{nd}}$ can be obtained by fitting the strain coefficients to Eq. (4). For the first order Fourier coefficients a two times larger fitting range (*L* < 0.5*R*_e_/*σ* |*μ*|; ***μ*** = ***g***·***b***) can be used than for the second order Fourier coefficients.

A third method can be used to obtain the value of ${\bar{P}}_{2\text{nd}}$ (and consequently ${\bar{P}}_{1\text{st}}$, cf. Eq. (9)). It follows from Eqs. (2), (4), (9), and (10):

$$4\;\ln[A_{1\text{st}}(L)]-\ln[A_{2\text{nd}}(L)]=3\;\ln[A^{S}(L)]-{\bar{P}}_{2\text{nd}}L^{2}\;\ln\;2.$$ (14)

Equation (14) is similar to the well-known Warren-Averbach equation: i.e.,

$$4\;\ln[A_{1\text{st}}(L)]-\ln[A_{2\text{nd}}(L)]=3\;\ln[A^{S}(L)].$$ (15)

The Warren-Averbach analysis obviously does not hold if Eqs. (4), (9), and (10) hold. Combining Eqs. (13) and (14) yields:

$${\bar{P}}_{2\text{nd}}L^{2}\;\ln\;2=2\;\ln[A_{1\text{st}}(L)]+\ln[A_{2\text{nd}}(L)]-6\;\ln[A_{2\text{nd}}\left(\frac{1}{2}L\right)].$$ (16)

Consequently, ${\bar{P}}_{2\text{nd}}$ can be obtained by plotting the right-hand side of Eq. (16) versus *L*^2^ and fitting a straight line.

**Fig. 1:**
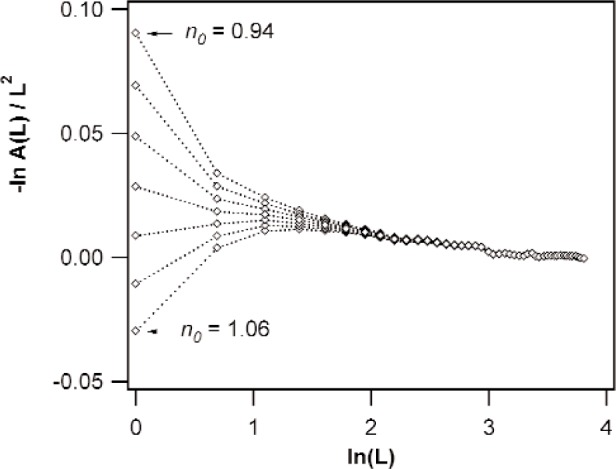
Krivoglaz-Wilkens plots of the ratio of {222} and {111} Fourier coefficients (corrected for instrumental broadening) of the Ni specimen annealed at 350 K. The different series are Krivoglaz-Wilkens plots of the same data that were first multiplied with (from top to bottom) *n*_0_ = 0.94, 0.96, 0.98, 1.0, 1.02, 1.04, and 1.06.

In the above paragraphs, a fit to Eq. (4), or similarly, to Eq. (11) was mentioned several times. For this purpose the so-called Krivoglaz-Wilkens plot [15] might be used. In that case ln[*A*^D^(*L*)]/*L*^2^ is plotted versus ln*L*. From the slope of the straight line, the value of ${\bar{P}}_{1\text{st}}$ (or ${\bar{P}}_{2\text{nd}}$) is obtained and the value of ${\bar{Q}}_{1\text{st}}$ (or ${\bar{Q}}_{2\text{nd}}$) follows from the intercept. However, a small experimental scaling error, induced by a small error in the first Fourier coefficient (cf. Sec. 3.1) already yields a large deviation from the expected straight line at low *L* values (see Fig. 1). The fit can therefore better be performed directly, allowing a small scaling error; i.e., in order to obtain *P* and *Q*, the data should be fitted to the function:

$$A^{D}(L)=n_{0}e^{-PL^{2}(Q-\ln\;L)},$$ (17)

where *n*_0_ is a constant close to one.

## 4. Experimental Illustration

**Fig. 2:**
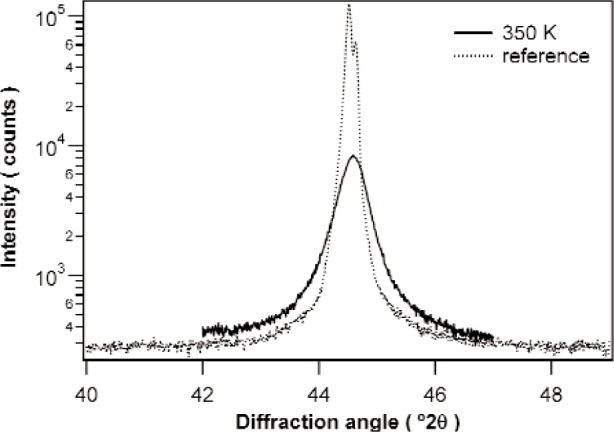
The {111} diffraction lines of the Ni specimen annealed at 350 K, and the Ni reference specimen.

As an example for the above methodology, the evolution of grain size, dislocation density and outer cutoff radius were investigated for a set of Ni layers on Si. The layers were simultaneously sputter deposited at room temperature to a thickness of 500 nm on seven oxidized <100> wafers. Transmission electron microscopy (TEM) experiments revealed a microstructure with more or less spherical grains with a diameter of about 20 nm. After deposition the specimens were subjected to anneals (0.5 h) at 300 K, 330 K, 350 K, 400 K, or 450 K, in gas mixture of Ar (volume fraction 95 %) and H_2_ (volume fraction 5 %) at 10^5^ Pa. A last specimen was annealed 2 h at 500 K. After annealing, the {111} and {222} diffraction line profiles were recorded for each specimen on a Bruker AXS D5005 *θ*-*θ* type diffractometer equipped with a diffracted-beam monochromator set to select Cu Kα radiation. In all cases, Bragg-Brentano geometry was used. The same reflections were recorded from a Ni reference specimen containing negligible structural broadening, using identical diffractometer settings. The reference specimen was produced by annealing Ni powder (diameter 2 μm). The broadness of the peaks obtained from the Ni reference specimen compare well to the broadness of the peaks obtained from our Al reference specimen, which shows slightly less broadening than the SRM660 LaB_6_ standard powder (see Ref. [16]). Figure 2 shows an example of the broadened 111 peak of the specimen annealed at 350 K, and the 111 peak of the reference specimen. For all peaks a linear background was subtracted before the Fourier coefficients were calculated. Instrumental broadening was removed by dividing the moduli of the Fourier coefficients of the Ni layer diffraction lines, by the moduli of the Fourier coefficients of the corresponding diffraction lines of the reference specimen (α_2_ correction was not applied). The thus obtained Fourier coefficients of the structurally broadened profiles were scaled such that the first Fourier coefficient (correlation distance zero) was equal to one.

**Fig. 3:**
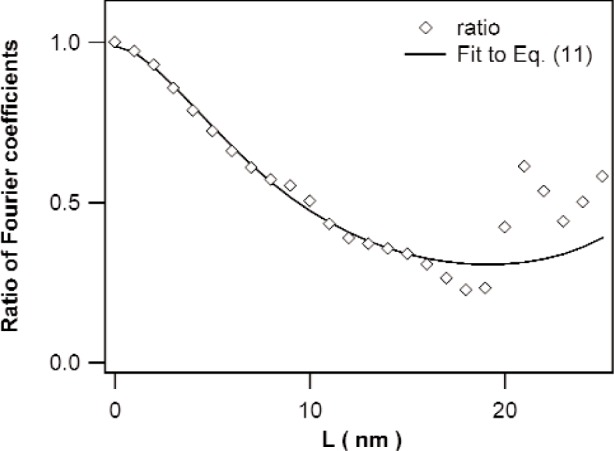
Ratio of the {222} and {111} Fourier coefficients (corrected for instrumental broadening) of the Ni layer annealed at 350 K. The solid line is a fit of Eq. (11) to the data belonging to correlation distances *L* up to 15 nm.

**Fig. 4:**
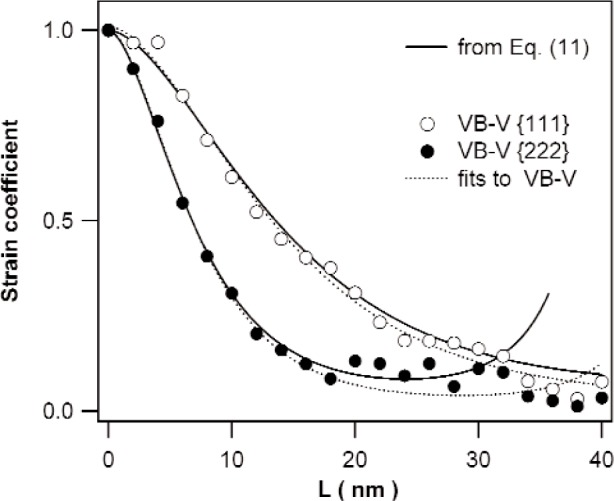
Strain Fourier coefficients of the {111} and {222} reflection of the Ni layer annealed at 350 K. Solid lines are the strain coefficients constructed from the values of ${\bar{P}}_{1\text{st}}$and ${\bar{Q}}_{1\text{st}}$, acquired from the fit in Fig. 3. Markers are the results of the VB-V analysis. Dotted lines are fits of Eq. (4) to the VB-V data, using the data belonging to correlation distances *L* up to 40 nm and 20 nm for the {111} and the {222} reflection, respectively.

**Fig. 5:**
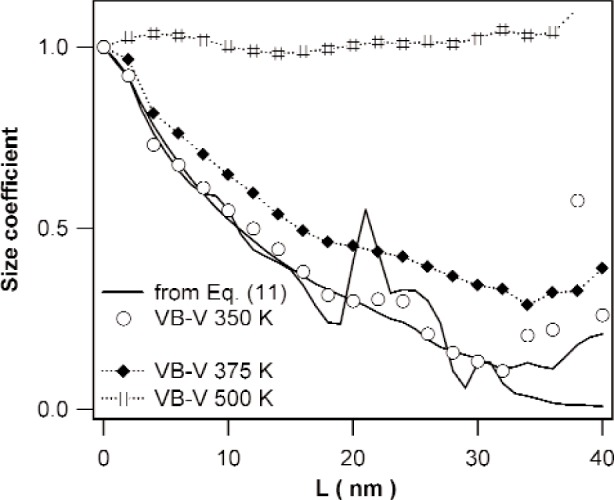
Size Fourier coefficients of the specimens annealed at 350 K, 375 K, and 500 K. The solid lines are the size coefficients for the specimen annealed at 350 K, obtained by dividing the Fourier coefficients of the {111} and {222} reflections by the strain coefficients that were constructed by fitting the ratio of the {111} and {222} profiles to Eq. (11), see Fig. 4. Markers (connected by dotted lines) are the result of the VB-V analysis.

The methods discussed in Sec. 3.2 are illustrated by means of the specimen annealed at 350 K. Fig. 3 shows the ratio of the Fourier coefficients of the {222} and {111} reflections together with a fit of Eq. (11) to the data. A good fit is obtained up to a correlation distance of about 15 nm. Using the values obtained for ${\bar{P}}_{1\text{st}}$ and ${\bar{Q}}_{1\text{st}}$, the strain and size coefficients can be determined. Figure 4 shows the first and second order strain coefficients constructed using Eqs (4), (9), and (10) (solid lines). Figure 5 shows the size coefficients, obtained by dividing the first and second order Fourier coefficients by the constructed strain coefficients (solid lines).

The size and strain coefficients were also determined with the VB-V analysis. In Fig. 5 the VB-V size coefficients, obtained using Eq. (13), are shown (open circles). The size coefficients determined with both methods agree well. Additionally the VB-V size coefficients for the specimens annealed at 375 K and 500 K are shown. Figure 4 shows the first and second order strain coefficients obtained with the VB-V analysis (circles). These strain coefficients were obtained by dividing the measured Fourier coefficients (after elimination of instrumental broadening) by the VB-V size coefficients. Again, good agreement exists between both methods of data analysis. Figure 4 also shows fits of Eq. (4) to the data (dotted lines).

**Fig. 6:**
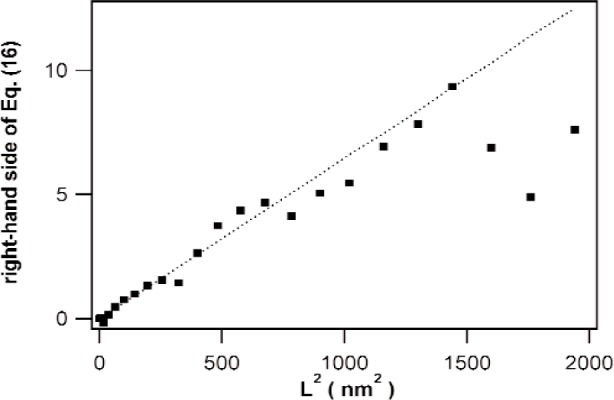
Right-hand side of Eq. (16): $2\;\ln[A_{1\text{st}}(L)]+\ln[A_{2\text{nd}}(L)]-6\;\ln[A_{2\text{nd}}\left(\frac{1}{2}L\right)]$ versus *L*^2^ and linear fit to the data (dotted line).

Figure 6 shows the plot proposed below Eq. (16) for the specimen annealed at 350 K, together with a fit to the data. The data follow reasonably the expected straight line. The value of ${\bar{P}}_{1\text{st}}$ was obtained from the slope of the fit, using Eq. (9).

**Fig. 7:**
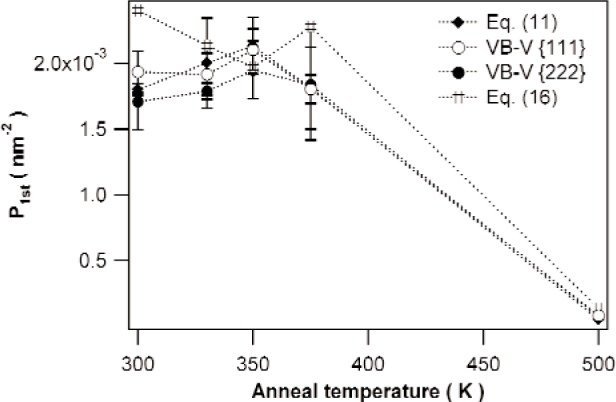
Values of ${\bar{P}}_{1\text{st}}$ for the specimens annealed at 300 K, 330 K, 350 K, 375 K, and 500 K, obtained from the fit of the ratio of the {222} and {111} Fourier coefficients to Eq. (11), the VB-V analysis on the {111} and {222} reflections, and the analysis on the basis of Eq. (16). For clarity, the error bars of the results using the method on the basis of Eq. (16) are omitted. The errors in these values are typically equally large as those observed for the other methods of data analysis.

**Fig. 8:**
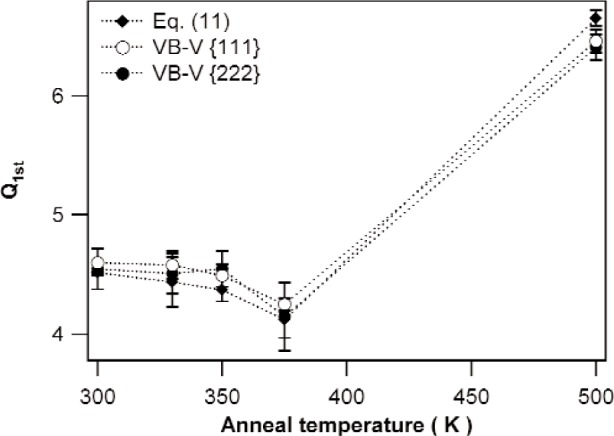
Values of ${\bar{Q}}_{1\text{st}}$ for the specimens annealed at 300 K, 330 K, 350 K, 375 K, and 500 K, obtained from the fit of the ratio of the {222} and {111} Fourier coefficients to Eq. (11), and the VB-V analysis on the {111} and {222} reflections.

Figures 7 and 8 show values for ${\bar{P}}_{1\text{st}}$ and ${\bar{Q}}_{1\text{st}}$, respectively, determined with the different methods of data analysis for the specimens annealed at 300 K, 330 K, 350 K, 375 K, and 500 K. For all specimens, the results of the different methods of data analysis correspond well. From the good agreement of the different methods of data analysis, we conclude that the assumption underlying the VB-V analysis, i.e., $\ln\left[A^{S}\left(\frac{1}{2}L\right)\right]=\frac{1}{2}\ln\left[A^{S}\left(L\right)\right]$ is valid in the present case.

Extrapolating the first few data of the size coefficients with a straight line to the correlation distance axis yields the so-called apparent grain size [7]. For the specimen annealed at 350 K, a value of about 15 nm is obtained (see Fig. 5), this value corresponds reasonably well with the grain size observed with transmission electron microscopy. Figure 5 shows that during annealing at 375 K some grain growth has occurred. The size coefficients for the specimen annealed at 500 K are close to one; i.e., for this specimen size broadening is negligible.

The dislocation density, represented by the value of ${\bar{P}}_{1\text{st}}$ (see Fig. 7), remains more or less constant as long as the anneal temperature does not exceed 375 K. In the specimen annealed at 500 K, the dislocation density has decreased considerably. The value of ${\bar{Q}}_{1\text{st}}$ (see Fig. 8), indicating the strain energy of a dislocation, decreases somewhat with temperature if the anneal temperature does not exceed 375 K. For the (recrystallized) specimen annealed at 500 K the value of ${\bar{Q}}_{1\text{st}}$ is much larger.

In summary, the following can be concluded about the evolution of microstructure of the nickel layers. Up to an annealing temperature of 375 K, relatively small changes in the microstructure of the specimens occur. In the specimen annealed at 375 K, the grains have grown out slightly (Fig. 5), and the strain energy of the dislocations (of which the density is constant) decreases somewhat with increasing temperature (i.e., decreasing ${\bar{Q}}_{1\text{st}}$ in Fig. 8). Consequently, the annealing temperatures not exceeding 375 K are high enough to allow for some dislocation rearrangement within the grains, but are too low to establish (large scale) recrystallization. In the specimen annealed at 500 K on the other hand, recrystallization has occurred. The specimen consists of large grains with low dislocation density. Because of the much lower dislocation density, the dislocation interaction is small and there is less possibility to minimize the strain energy by means of dislocation rearrangement. Therefore, the outer cut-off radius for this specimen, reflected by the value of ${\bar{Q}}_{1\text{st}}$, is relatively large.

For a quantitative interpretation of the values of ${\bar{P}}_{1\text{st}}$and ${\bar{Q}}_{1\text{st}}$, determined in the above analyses, in terms of dislocation distribution parameters as dislocation density and outer cut-off radius, knowledge of the contrast factor is necessary. The determination of the contrast factor is in general a difficult task and is beyond the scope of the present paper. In Refs. [5,11,17] suggestions are given how contrast factors can be obtained experimentally for heavily textured specimens and specimens with a homogeneous dislocation distribution. For the present data, assuming that only screw dislocations $\left(\text{Burgers vector}\;\frac{1}{2}<110>\right)$ are present, it holds that *C*_i_, for the dislocations with Burgers vectors inclined to the diffraction vector and for the dislocations with Burgers vectors perpendicular to the diffraction vector equal 0.1384 and 0, respectively [17]. Assuming that dislocations are distributed homogeneously over the different sets and that for the different sets the outer cut-off radius is equal it holds: ${\bar{P}}_{1\text{st}}=\frac{1}{2}\cdot 0.1384\cdot \frac{\pi }{2}g^{2}b^{2}\rho_{\text{tot}}$, where *ρ*_tot_ is the total dislocation density, and $R_{e}=\exp[{\bar{Q}}_{1\text{st}}-2\;\ln\;2+\frac{1}{3}+\ln(\frac{1}{3}\sqrt{3})]$. It follows that the dislocation densities and outer cut-off radii, before recrystallization, are about 10^16^ m^–2^ and 18 nm, respectively. After recrystallization these values are about 2 × 10^14^ m^–2^ and 150 nm. It is stressed here that these values should be considered as rough estimates. Further, it should be noted that dislocations might be generated during cooling down. Due to the different thermal expansion coefficients of silicon and nickel a thermal (tensile) strain develops in the nickel layer during cooling down. For the specimen annealed at 500 K, this strain is approximately 0.2 %. X-ray diffraction strain measurements revealed that the strain in the nickel layer annealed at 500 K, at room temperature is close to this value, which suggests that the thermal strains are hardly plastically relaxed, and consequently, not many dislocations are formed during cooling down. Similar experiments to thin Al layers in which plastic deformation was observed during cooling down revealed dislocation densities up to 3 × 10^14^ m^–2^ [4,5]. Thus, in this case, possible dislocations formed during cooling down can be neglected for the layers annealed at temperatures up to 375 K, that show much larger dislocation densities. However, for the specimen annealed at 500 K, it cannot be excluded that the dislocations are (in part) generated during cooling down.

A last example concerns the limitations of the above analyses. For the nickel layers treated above it has been assumed that a (more or less) homogeneous grain size and dislocation distribution was present in the specimens. However, the specimens annealed at 400 K and 450 K have partly recrystallized. The microstructure of these specimens therefore consists of a mixture of small grains with large dislocation density (i.e., comparable to the not yet recrystallized specimen annealed at 375 K) and large recrystallized grains (comparable to the grains in the specimen annealed at 500 K) with low dislocation density. For such inhomogeneous specimens, the methods treated above are not useful.

**Fig. 9:**
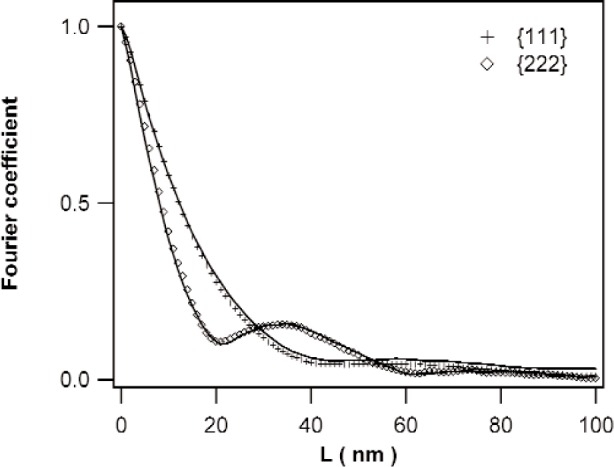
Fourier coefficients (not corrected for instrumental broadening) of the {111} and {222} reflections of a nickel layer annealed at 400 K. Solid lines are the sum of 0.54 times the Fourier coefficients of the specimen annealed at 375 K and (1-0.54) times the Fourier coefficients of the specimen annealed at 500 K.

In this case, an analysis of the diffraction line broadening is still possible. The microstructures of the specimens annealed at 400 K and 450 K are considered to be a mixture of the microstructures of the specimens annealed at 375 K and 500 K. Then, the diffraction peaks of the specimens annealed at 400 K and 450 K are simply the sums of the diffraction peaks of the diffraction peaks of the specimens annealed at 375 K and 500 K, scaled with their respective (diffracting) volume fractions. The same holds for the Fourier coefficients of the diffraction peaks. Thus, the volume fractions of the “375 K microstructure” and the “500 K microstructure” can be obtained by fitting the volume fractions such, that shape the (Fourier coefficients of the) measured peak corresponds to the weighted sum obtained from (Fourier coefficients of) the 375 K and 500 K peaks. Applying this method to the Fourier coefficients, “375 K” volume fractions of 0.57(4) and 0.40(4) were obtained for the specimens annealed at 400 K and 450 K, respectively. In Fig. 9 the Fourier coefficients of the {111} and {222} reflection of the specimen annealed at 400 K are shown, together with the weighted sum of the Fourier coefficients of the specimens annealed at 375 K and 500 K. The specimen can quite well be characterized as a mixture of the microstructures before and after recrystallization. Note that for this procedure, neither correction for instrumental broadening, nor determination of the strain broadening was necessary, in contrast to the methods above. Therefore, possible errors made by the removal of the instrumental broadening are avoided.

In the last example it was possible to perform a diffraction line shape analysis despite the inhomogeneity of the specimens under consideration. In general however, this may not be possible and investigations to dislocation distributions using x-ray line profile analysis, for inhomogeneous microstructures may become very difficult.

## 5. Conclusions

Several methods can be used to determine dislocation distribution parameters from diffraction line broadening measurements in specimens for which both strain broadening caused by dislocations and size broadening occurs. If the strain broadening can be described with the model due to Wilkens, dislocation distribution parameters can be determined from the ratio of the Fourier coefficient of diffraction line profiles from two orders of reflection, as well as using the Van Berkum-Vermeulen analysis. The use of the Warren-Averbach method is in this case dissuaded.

For thin nickel layers on silicon, the analysis on the basis of the ratio of the Fourier coefficients of two orders of reflection and the Van Berkum-Vermeulen analysis yield equal results within experimental precision.

Annealing thin nickel layers with large dislocation density and small grain size, at temperatures up to 375 K does not lead to large scale grain growth and changes in dislocation density. However, the outer cut-off radius decreases somewhat, which suggests that strain energy is minimized at these temperatures by means of movement of the dislocations within the grains. Annealing at 400 K and 450 K leads to an inhomogeneous microstructure that consists of large grains with low dislocation density and small grains with high dislocation density. Complete recrystallization occurs during annealing at 500 K; after annealing the specimen consists of large grains with low dislocation density.

## Footnotes

1 More generally, if the Fourier coefficients of any two line profiles I and II, with exactly the same size broadening and of which the strain broadening is well described by the Wilkens expressions, are divided it holds:

$$\frac{A^{\mathrm{II}}(L)}{A^{I}(L)}=\frac{A^{D(\mathrm{II})}(L)}{A^{D(I)}(L)}=\exp\left\{-({\bar{P}}^{\mathrm{II}}-{\bar{P}}^{I})L^{2}\left(\frac{{\bar{P}}^{\mathrm{II}}{\bar{Q}}^{\mathrm{II}}-{\bar{P}}^{I}{\bar{Q}}^{I}}{{\bar{P}}^{\mathrm{II}}-{\bar{P}}^{I}}-\ln\;L\right)\right\}$$

Consequently, the ratio of any two line profiles that can be written according to Eq. (4) also obeys Eq. (4).
